# Diagnostic and Prognostic Risk Assessment of Heat Shock Protein *HSPA1B* rs2763979 Gene Variant in Asthma

**DOI:** 10.3390/genes13122391

**Published:** 2022-12-16

**Authors:** Salwa Faisal, Sherouk Abdelaal, Mohammed A. Jeraiby, Fatihi Hassan Soliman Toaimah, Shahad W. Kattan, Abdelhady Ragab Abdel-Gawad, Eman Riad, Eman A. Toraih, Manal S. Fawzy, Ahmed Ibrahim

**Affiliations:** 1 Department of Medical Biochemistry and Molecular Biology, Faculty of Medicine, Suez Canal University, Ismailia 41522, Egypt; 2 Department of Pediatrics, Faculty of Medicine, Suez Canal University, Ismailia 41522, Egypt; 3 Department of Biochemistry, Faculty of Medicine, Jazan University, Jazan 82621, Saudi Arabia; 4 Division of Pediatric Emergency Medicine, Department of Pediatrics, Hamad Medical Corporation, Doha 3050, Qatar; 5 Department of Clinical Pediatrics and Clinical Emergency Medicine, Weill Cornell Medicine-Qatar, Doha 24144, Qatar; 6 Department of Clinical Academic Education, College of Medicine, Qatar University, Doha 2713, Qatar; 7 Department of Medical Laboratory, College of Applied Medical Sciences, Taibah University, Yanbu 46423, Saudi Arabia; 8 Department of Clinical and Chemical Pathology, Faculty of Medicine, Sohag University, Sohag 82524, Egypt; 9 Department of Chest Diseases and Tuberculosis, Faculty of Medicine, Suez Canal University, Ismailia 41522, Egypt; 10 Division of Endocrine and Oncologic Surgery, Department of Surgery, Tulane University School of Medicine, New Orleans, LA 70112, USA; 11 Medical Genetics Unit, Department of Histology and Cell Biology, Suez Canal University, Ismailia 41522, Egypt; 12 Department of Biochemistry, Faculty of Medicine, Northern Border University, Arar 1321, Saudi Arabia

**Keywords:** asthma, *HSPA1B*, propensity-matched analysis, pulmonary function tests, real-time PCR, rs2763979, single nucleotide polymorphism

## Abstract

Given the significant role the heat shock protein Hsp70 plays in modulating cellular homeostasis in several chronic inflammatory disorders, the genetic variation of the inducible *HSP70* (*HSPA1B*) gene may impact protein expression and disease phenotype. The *HSPA1B* rs2763979 variant has been associated with multiple inflammatory scenarios, but no previous studies have explored its association with asthma. In this sense, this cross-sectional study enrolled 90 children with asthma and 218 age-/sex-matched healthy volunteers for rs2763979 variant genotyping by TaqMan allelic discrimination analysis. The results were investigated under several genetic models and associated with disease susceptibility and clinicolaboratory data. Overall analysis, including the 308 participants, revealed a higher C allele frequency among patients relative to controls (43.0% vs. 33%, *p* = 0.006). Furthermore, patients with the C variant initially had a higher risk of asthma under heterozygous (OR = 2.75, 95%CI = 1.46–5.18, *p* = 0.003), homozygous (OR = 3.35, 95%CI = 1.19–9.39, *p* = 0.008), dominant (OR = 2.83, 95%CI = 1.52–5.25, *p* < 0.001), and overdominant (OR = 2.12, 95%CI = 1.20–3.74, *p* = 0.008) models. However, after employing a 1:1 nearest propensity matching analysis, the studied variant showed only borderline significance with asthma under the dominant model in 71 matched cohorts. Interestingly, patients who carry the rs2763979 CC genotype showed favorable spirometric parameters in terms of better (mean ± SD) forced vital capacity (86.3 ± 7.4 vs. 77.7 ± 6.1 and 75.7 ± 7.2 for CT and TT, respectively, *p* = 0.021), forced expiratory volume in one second before bronchodilation (60.7 ± 12.9 vs. 54.9 ± 7.6 and 56.1 ± 7.5 for CT and TT, respectively, *p* = 0.021), and an improvement in peak expiratory flow rate after inhaled salbutamol bronchodilator (*p* = 0.044) relative to the counterpart genotypes. In conclusion, the *HSPA1B* rs2763979 variant might have prognostic utility as a genetic marker for asthma in our population. Further larger studies on different ethnicities are recommended to validate the results.

## 1. Introduction

Asthma is a chronic inflammatory lung disorder characterized by bronchial hyper-reactivity to various stimuli such as infections, allergens, and environmental irritants with reversible bronchial obstruction and gradually progressive structural remodeling. It frequently affects children, being one of the most common chronic illnesses among them, with a global mortality rate ranging from 0 to 0.7 per 100,000 [1].

The exact etiology of asthma remains unclear; however, genetic and environmental risk factors contribute virtually to asthma pathogenesis. Identifying such genes and molecular pathways will be essential for predicting disease outcomes and establishing proper therapeutic approaches [2].

Asthma is a chronic bronchi inflammation with diverse phenotypes categorized into allergic and non-allergic or recently “T2-high” and “Non-T2” subtypes with evident implications for several interleukins in the disease etiopathology. Furthermore, some damage-associated molecular pattern (DAMP) molecules, such as the 70 kDa heat shock proteins (HSP70), were reported to play a pivotal role in asthma [3].

**Figure 1:**
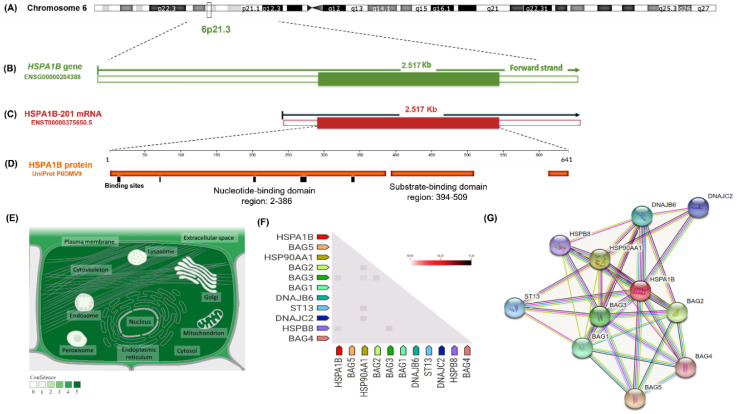
Structural features of heat shock protein family A (Hsp70) member 1B (*HSPA1B*) gene and protein. (**A**) The gene (Gene ID: 3304) encoding a 70 kDa protein, a member of the heat shock protein 70 family, is located on the short arm of chromosome 6-NC_000006.12. (**B**) The gene is 2517 bp long, spanning from position 31,827,738 to 31,830,254 (*Homo sapiens* assembly; GRCh38.p14) along the forward strand. It consists of a single coding exon and lacks introns. (**C**) The gene has a single transcript of 2517 nucleotides, including the 5′- and 3′-untranslated regions (UTRs). (**D**) The encoded protein is a single polypeptide chain of 641 amino acid residues. The nucleotide-binding domains are shown in orange, and the binding sites are shown as small black boxes by which the protein goes through repeated cycles of ATP binding/hydrolysis and nucleotide exchange and release. (**E**) The subcellular localization of the HSPA1B. The degree of green darkness correlates with abundance. (**F**) The triangle matrix of the observed gene coexpression of HSPA1B in humans. The color intensity shows the confidence level of association, given the overall expression data in the organism. HSPA1B shows coexpression with the molecular chaperone regulator 3 (BAG3), which has anti-apoptotic activity and acts as “a nucleotide-exchange factor promoting the release of ADP from the HSP70, thereby triggering substrate release” (score = 0.985). In addition, HSPA1B is coexpressed with a high level of confidence with the heat shock protein β-8 (HSPB8), which displays “temperature-dependent chaperone activity” and belongs to the HSP20 family (score = 0.956). (**G**) A protein–protein interaction network illustrates the predicted functional partners of the HSPA1B. BAG1/2/3/4/5: “BAG family molecular chaperone regulators act as nucleotide-exchange factors (NEFs) promoting the release of ADP from the HSP70 and HSC70 proteins thereby triggering client/substrate protein release”, HSP90AA1: HSP 90-α, “a molecular chaperone that promotes the maturation/structural maintenance and proper regulation of specific target proteins involved in cell cycle control and signal transduction”, DNAJB6: “DNAJ homolog subfamily B member 6 that plays an indispensable role in the organization of KRT8/KRT18 filaments”, DNAJC2: “DNAJ homolog subfamily C member 2 that acts both as a chaperone in the cytosol and as a chromatin regulator in the nucleus, ST13: “Hsc70-interacting protein”. Data sources: https://www.genecards.org/, https://www.ncbi.nlm.nih.gov/gene/3304, https://asia.ensembl.org/Homo_sapiens/Transcript/, https://www.uniprot.org/, https://compartments.jensenlab.org/, https://string-db.org/ (accessed on 5 October 2022).

HSP70 is a family of molecular chaperones that represents the most ubiquitous and highly conserved chaperones, which take part in cellular proteostasis as protein folding, importing, and assembly, which augment cellular survival and prevent damage to other cochaperones [4] (Figure 1). HSP70 is expressed constitutionally, except for HspA1A, HspA1B, and HspA5, which are stress-inducible [5]. It has been identified as a potential biomarker or an immunomodulant linked to different pathologies such as inflammation, malignancy, fibrosis, and autoimmunity [6,7].

In asthma, extracellular HSP70 proteins have emerged as pro-inflammatory mediators, being abundantly expressed in the serum, sputum, and bronchoalveolar lavage fluid of asthmatic patients [8]. Positive regulation of airway inflammation and goblet cell hyperplasia was further confirmed in “soluble egg antigen-induced allergic asthma” in mice via Th2-response modulation [9]. On the other hand, Shevchenko et al. described HSP70 proteins as anti-inflammatory mediators [10], concluding their divergent role and necessitating further studies to confirm.

A growing body of evidence points to HSP70′s role in asthmatic inflammation; nevertheless, the genetic variants associated with this inflammatory marker still need further research. The *HSP70* (*HSPA1B*) gene (Gene ID: 3304) single nucleotide polymorphisms (SNPs) were found to be risk factors in several human disorders, including sarcoidosis [11], ulcerative colitis/Crohn’s disease [12], systemic lupus erythematous [13], and diabetes-related kidney diseases [14,15], among others. The *HSPA1B* rs2763979 variant (c.+1538A>G) is located on Chr6: 31,826,815 (on Assembly GRCh38) and has been associated with chronic obstructive lung diseases and lung cancer [16,17], but no previous studies have explored its association with asthma in particular in the Middle East population. Therefore, this work was conducted to assess the association of the *HSPA1B* rs2763979 variant with the risk of asthma and disease characteristics aiming at prognostic assessment.

## 2. Materials and Methods

### 2.1. Study Design and Population

This comparative cross-sectional study included (1) ninety children with asthma diagnosed according to the “Global Initiative for Asthma (GINA)” [18]. Patients aged 6 to 18 years were recruited from the Pediatric Allergy and Immunology clinic and the Chest Diseases and Tuberculosis department, Suez Canal University Hospitals, Ismailia, Egypt, from April 2018 to August 2019. (2) A control group of 218 age-and sex-matched healthy volunteers without any previous history of medications or any health problems at the time of the study attended the outpatient clinics for non-medical reasons.

The following patients were excluded from the study: asthmatic children with chronic comorbidities, recent symptoms of acute respiratory infection, recent use of systemic steroids, or non-compliance with therapy. Detailed histories and examinations of all participants were recorded, including demographic data, the duration of asthma, onset and frequency of symptoms, asthma phenotype, and triggering factors. Based on GINA recommendations, we evaluated comorbidities, level of asthma control, disease severity, and treatment adherence.

Body mass index (BMI) was calculated as body weight (kg) divided by height (in meters) squared [19]. Based on the Tanner stages, pubertal development was assessed [20]. This study was approved by the Ethics Committee of the Faculty of Medicine, Suez Canal University, Egypt. The Declaration of Helsinki guidelines were followed, and informed consent was obtained from all subjects/their parents involved in the study before taking part.

### 2.2. Pulmonary Function Tests

Spirometry was performed using the Medisoft ExpAir spirometry (Viasys Healthcare, Conshohocken, PA, USA) to assess the basal pulmonary function tests following the American Thoracic Society (ATS) and European Respiratory Society (ERS) recommendations [21]. The peak expiratory flow rate (PEFR), the forced vital capacity (FVC), and the forced expiratory volume in one second (FEV1) were all measured. A 400 µgm dose of salbutamol (Ventolin; GlaxoSmithKline) was given with a metered-dose inhaler and spacer device. Post-bronchodilator forced spirometry was performed 15 min after salbutamol administration. Response to salbutamol was calculated with the following equation: [BDRBASE = ((postbronchodilator FEV1 − prebronchodilator FEV1)/prebronchodilator FEV1) × 100] [22].

### 2.3. Methacholine Challenge Test (MCT)

A methacholine challenge test was performed to assess the degree of airway hyperresponsiveness according to the American Association for Respiratory Care (AARC) clinical practice guidelines [23]. The amount of methacholine (in mg/mL) required to produce a 20% reduction in FEV1 was reported. Positive airway hyperresponsiveness is defined as a PC20 value < 8 mg/mL [24].

### 2.4. Laboratory Investigations

Venous blood samples were collected in EDTA and plain tubes. The absolute peripheral blood eosinophil count was assayed in the tubes by Coulter count (Beckman Coulter Ltd., Brea, CA, USA) with the microscopic manual differential count. An eosinophilic count above 400 cells/mm^3^ was considered absolute eosinophilia [25]. Total serum IgE was measured using an enzyme-linked immunosorbent assay (ELISA) (AccuBind^®^ ELISA, Monobind Inc., Lake Forest, CA, USA). IgE is considered to be high if the total IgE level is greater than or equal to 100 IU/mL [26].

### 2.5. HSPA1B rs2763979 Allelic Discrimination Analysis

Genomic DNA was extracted from peripheral venous blood using a QIAamp DNA Blood Mini kit (Cat. No. 51104, QIAGEN, Hilden, Germany) according to the vendor’s guidelines. The concentration and purity of isolated nucleic acids were evaluated by a Nanodrop-1000 spectrophotometer (NanoDrop Tech., Wilmington, USA), then stored at −80 °C till the time of allelic discrimination analysis. The intronic rs2763979 variant genotyping was carried out using a TaqMan assay following the manufacturer’s protocols. The assay (C___3052606_10; Catalog #: 4351379, Applied Biosystems, Foster City, CA, USA) contains specified probes (VIC/FAM) to determine the wild/mutant alleles, respectively, in the context sequence: ACTGTGAGGTCCTACTTCTACACAC[C/T]GTCCAGGAGTGAACCAGGAATTGAG, according to the build GRCh38. The components/concentrations of each PCR run were mentioned in detail previously [27]. The PCR was carried out on a StepOne™ Real-Time PCR System (Applied Biosystems) through a blinded protocol regarding the case/control status. The PCR program started with 10 min of initial denaturation at 95 °C, followed by 40 amplification cycles for 15 s at 95 °C and annealing for 1 min at 60 °C, then a final step for 30 s at 60 °C [28]. Negative controls were tested with each run. Ten percent of all participants were randomly re-genotyped in separate runs with a concordance rate of 100%. Analysis for post-amplification data was carried out by SDS software (v1.3.1., Applied Biosystems).

### 2.6. Statistical Analysis

SPSS version 27.0 (IBM Corp. Armonk, NY) was applied for statistical data analysis. G*Power (version 3.0.10) was applied to calculate the study power. The estimated power was 84% at total sample size = 142, calculated effect size = 0.5, and α error probability = 0.05. *HSPA1B* rs2763979 allele/genotype frequencies were calculated as previously described [29]. A Chi-square test was used for comparison. The online Encyclopedia for Genetic Epidemiology (OEGE) software (http://www.oege.org/software/hwe-mr-calc.shtml, accessed on 25 August 2022) was used for the estimation of Hardy–Weinberg equilibrium (HWE). Logistic regression models were carried out for the calculation of odds ratios (OR) with a 95% confidence interval (CI) under various genetic association models [30]. Propensity score analysis was carried out using the nearest neighbor method with a ratio of 1:1 via the MatchIT R package. Association of the *HSPA1B* rs2763979 SNP with the clinicolaboratory features was performed using two-sided Chi-square, one-way ANOVA, and Kruskal–Wallis tests. A two-tailed *p*-value of 0.05 was considered statistically significant.

## 3. Results

### 3.1. Baseline Characteristics of the Study Population

A total of 308 (90 patients and 218 controls) participants (aged 6–18 years) was initially included in this study. There were no significant differences between the two study groups regarding age and sex (Table 1). A significantly higher frequency of positive family history for asthma was observed in the initial patient cohort (27.8% vs. 7.3%; *p* < 0.001), but this difference disappeared by employing the propensity-matched analysis, as seen in Table 1.

### 3.2. Genotype and Allele Frequencies of HSPA1B rs2763979 T>C Polymorphism

Genotype frequency in controls followed Hardy–Weinberg equilibrium (*p* = 0.064). Their minor allele frequency (C allele) was 0.33. The C allele was more frequent in patients with asthma (43.0% vs. 33%, *p* = 0.006). Similarly, the C/T–C/C genotypes predominated among patients (77.0% vs. 58%), while T/T genotype homozygosity was more represented in controls (42%) compared to patients with asthma (23%) (*p* = 0.008) (Table 2).

### 3.3. Association of HSPA1B rs2763979 Polymorphism with Asthma Risk

Analysis of rs2763979 polymorphism under different genetic association models among patients with asthma (n = 90) and controls (n = 218) showed that patients with the C variant had a higher risk of asthma under heterozygous comparison (C/T vs. T/T: OR = 2.75, 95%CI = 1.46–5.18, *p* = 0.003), homozygote comparison (C/C vs. T/T: OR = 3.35, 95%CI = 1.19–9.39, *p* = 0.008), dominant model (C/T–C/C vs. T/T: OR = 2.83, 95%CI = 1.52–5.25, *p* < 0.001), and overdominant model (C/T vs. T/T–C/C: OR = 2.12, 95%CI = 1.20–3.74, *p* = 0.008) (Table 3).

**Figure 2:**
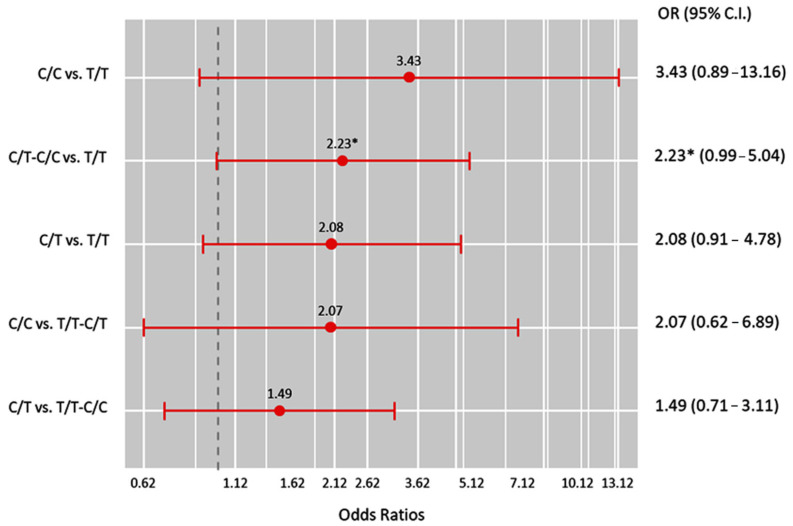
Genetic association models for propensity-matched cohorts (each = 71). OR (95% C.I.): odds ratio (95% confidence interval). * *p*-value < 0.05.

After employing a 1:1 nearest propensity matching analysis, 71 paired matched cohorts were used for further analysis (Figure 2). Surprisingly, the studied variant showed only borderline significance with asthma susceptibility under the dominant model (C/T–C/C vs. T/T: OR = 2.23, 95%CI = 0.99–5.04, *p* = 0.049).

### 3.4. Association of HSPA1A rs2763979 Genotypes with Demographic and Clinicolaboratory Data in Patients with Asthma

The studied variant was not associated with the demographic, clinical, or laboratory data in the patient group, but the CT genotype carriers were more likely to have a history of food allergy (*p* = 0.031) (Table 4).

### 3.5. Association of HSPA1A rs2763979 Genotypes with Disease Control, Treatment Response, and Spirometric Parameters in Patients with Asthma

Table 5 shows that the studied variant was not associated with the degree of asthma control or the therapeutic levels in the patient cohort (*p* > 0.05). However, patients who carried the rs2763979 CC genotype showed favorable spirometric parameters in terms of better (mean ± SD) forced vital capacity (86.3 ± 7.4 vs. 77.7 ± 6.1 and 75.7 ± 7.2 for CT and TT, respectively, *p* = 0.021), (mean ± SD) forced expiratory volume in one second before bronchodilation (60.7 ± 12.9 vs. 54.9 ± 7.6, and 56.1 ± 7.5 for CT and TT, respectively, *p* = 0.021), and an improvement in peak expiratory flow rate after inhaled salbutamol bronchodilator (*p* = 0.044) than the counterparts genotypes.

## 4. Discussion

Asthma is a devastating respiratory disease with high mortality and morbidity rates. Genetic predisposition plays a crucial role in asthma development, with heritability estimates varying between 35% and 95% [31]. Several genetic studies, including genome-wide association and candidate-gene linkage studies, have identified numerous asthma susceptibility genes [32,33,34,35]. One gene family widely investigated is HSP70 [36,37,38].

The *HSP70* gene variants have been identified as risk factors involved in the pathogenesis of numerous human diseases, mainly immune-mediated, such as sarcoidosis [11], multiple sclerosis [39], and inflammatory bowel disease [12]. HSP70 chaperones are essential for housekeeping processes as well as antigen presentation and immune response, including both innate and adaptive categories. Accumulating evidence has shed light on the potential value of HSP70 in the pathogenesis of asthma. In this sense, this study aimed to unravel the association of the *HSPA1B* rs2763979 variant with asthma susceptibility and disease characteristics.

We found a substantial difference in both allelic and genotypic distribution of the rs2763979 variant between the study groups. Briefly, the frequency of the C allele was higher in the patients’ groups relative to the controls. Overall analysis showed that the *HSPA1B* rs2763979 variant conferred a risk of asthma under codominant, dominant, overdominant, and log-additive models. However, after employing the propensity-matched analysis to control the confounders and yield 1:1 matching between the two cohorts (n = 71), the significant association of *HSPA1B* rs2763979 with BA susceptibility was overlooked under all genetic models except the dominant model with borderline significance. These findings could emphasize the importance of running rigorous, well-controlled, and confounder-free analyses while testing the genotype–phenotype associations related to population traits. Additionally, asthma is one of the complex disorders that is not influenced only by genetic factors but by several gene–environment interactions that should be further elucidated [40].

To the authors’ knowledge, this study is the first to explore the genetic association of *HSPA1B* rs2763979 with asthma. A previous study deciphered the role of another regulatory variant (rs1061581) in pediatric asthma, and no association was detected [38]. In a closely related respiratory disorder, chronic obstructive pulmonary disease (COPD), *HSPA1B* rs2763979 (CT + TT) was associated with disease susceptibility together with another promoter SNP (rs6457452), highlighting their possible implication in chronic respiratory diseases susceptibility [16]. Similarly, this variant was significantly associated with SLE risk as a single allele or haplotype model with other *HSPA1L* and *HSPA1A* variants (rs2075800, rs2227956, rs1043618, rs3115673) and correlated with the production of autoantibodies to Ro and La [13]. Additionally, *HSPA1B* rs2763979 showed a significant association with the risk of diabetic nephropathy in type 2 diabetes among the Indian population [14]. Of note, the studied variant was widely examined in noise-induced hearing loss with contradictory results, as Li et al. [41] found the TT genotype conferred a risk of the disease, while the meta-analysis done by Zong et al. declared no significant association [42]. In the case of coronary artery diseases, no significant association was reported [43]. Regarding the neoplasms, this variant was assessed with lung cancer, in which the TT genotype was associated with poor prognosis and decreased gene expression in both normal bronchial epithelial and malignant cancer cells [17].

**Figure 3:**
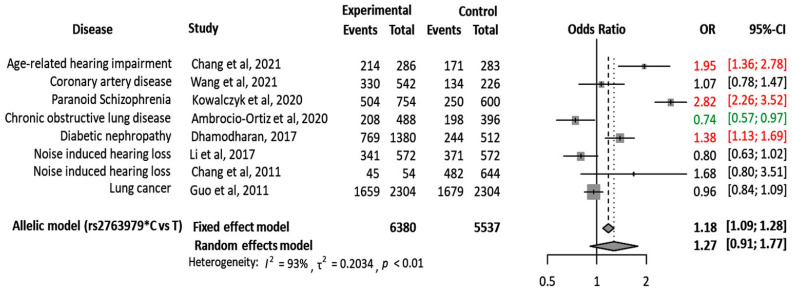
Forest plot of the pooled data for the association between *HSPA1B* rs2763979 variant and different disorders under the allelic model stratified by the type of the disorder [14,16,17,41,43,46,47,48]. Red text indicates significant increased risk of association, while green text indicates significant protective association with the disease, OR: odds ratio, CI: confidence interval. The meta-analysis was carried out using the Comprehensive Meta-analysis package version 3.0 (Biostat, Englewood, NJ, USA).

In an attempt to reach an evidence-based conclusion, the authors reviewed the study variant-related publications from which genotype count in cases and controls could be obtained (Table S1). Eight articles on different human diseases [14,16,17,41,43,44,45,46] with complete genetic data were enrolled. Our meta-analysis using the random effects model revealed that patients with the rs2763979*C allele were more likely to develop paranoid schizophrenia (OR = 2.82, 95%CI = 2.26–3.52), age-related hearing impairment (OR = 1.95, 95%CI = 1.36–2.78), and diabetic nephropathy (OR = 1.38, 95%CI = 1.13–1.69). In contrast, the C variant conferred protection against chronic obstructive lung disease (OR = 0.74, 95%CI = 0.57–0.97) (Figure 3). Such contradictions (risky vs. protective phenotype) in the previous studies could be explained by (1) the acknowledged genetic heterogeneity among different study populations, (2) the differences in environmental factors and geographical distribution, (3) variation in the sample size and study methodology, and (4) the underlying effects which the genetic variants induce in “a cell-type-specific and context-dependent manner” [44,45].

It is worth noting that rs2763979 is located in the *HSPA1B* 5′ flanking region that is non-coding but plays an essential role in controlling gene expression. Additionally, *HSPAIB* occurs within the “class III *MHC*” region that is highly pleomorphic and contains strong linkage disequilibrium (LD). The studied variant was experimentally identified in LD with HLA alleles, particularly HLA-DRB1*03. Therefore, such SNP or others present in LD might associate with alteration in gene expression and impact disease association and/or outcomes [49]. Notably, aberrant expression of *HSP70* has been evidenced in patients with asthma. Several studies demonstrated upregulation of the *HSPA1B* gene and protein in asthmatic sputum and airway epithelial cells, in addition to the high circulating level correlated to disease severity and asthmatic symptom score, highlighting its potential role as a diagnostic and/or prognostic marker for asthma [8,50,51,52].

HSP70 proteins were conveyed to have pro-inflammatory and anti-inflammatory effects in airway inflammation that might be related to the protein characteristics. While intracellular HSP70 are cytoprotective chaperones, the extracellular forms (eHSP) serve as alarmins and possess pro-inflammatory properties, and both forms are elevated in asthma, contributing substantially to allergic and non-allergic subtypes [8,53]. Additionally, interacting receptors could be another determent, as HSP70 stimulation through Siglec-5 acts as an anti-inflammatory signal, while stimulation through Siglec-14 is pro-inflammatory [3,54]. Mechanistically, eHSP70 effects might be mediated via (1) engaging proper receptors such as toll-like receptors-2/-4, the cluster of differentiation 14, the lectin-like oxidized low-density lipoprotein-1, and the receptor for advanced glycation end-products to activate immune responses [3,55]; and (2) binding to antigenic peptides and delivering them to antigen-presenting cells [56].

In allergic asthma, the effects of eHSP70 are paradoxical. Yombo et al. demonstrated that hematopoietic, not epithelial HSP70, activated Th2 and type 2 cytokine production as IL-4, IL 5, and IL-13, leading to the maintenance of a pro-allergic response, including eosinophilia, airway inflammation, goblet cell hyperplasia, and mucus hypersecretion [9]. At the same time, loss of HSP70 could ameliorate the manifestation of allergic asthma and suppress the Th2 immune response in the SEA-challenged model [9]. On the contrary, Shevchenko et al. indicated exogenously supplied HSP70, down-regulated eosinophilia, the allergen-specific IgE, and the pro-allergic cytokines through a neutrophil-dependent mechanism [10]. Notably, HSP70 proteins facilitate the recruitment of neutrophils known to block Th2 proliferation and prevent ILC2 function and monocyte–dendritic cell antigen presentation [57]. Moreover, the HSP70/CD80 DNA vaccine has been found to reduce airway remodeling in chronic asthma via regulating the transcription factors T-bet and GATA-3, which are essential for Th1 and Th2 differentiation [58].

Regarding the association of the studied rs2763979 variant with disease characteristics, the current results showed no association with disease severity or laboratory data. These findings could be attributed to a relatively limited sample size after employing the propensity-matched analysis. Additionally, asthma is a multifaceted disease with multiple and complex interacting factors contributing to disease severity [59]. In this line, no association was demonstrated between the same variant and COPD [16]. On the other hand, the studied variant was associated with one of the disease triggers, such as food allergy. Several HSP70 members are described as allergen-promoting allergic airway diseases in sensitive individuals. Additionally, cross-reactivity to self-HSP70 was evidenced in atopic dermatitis patients [60]. A further large-scale study should be warranted to unleash such relations and underlying molecular mechanisms. In concern to the pulmonary function tests, the current variant showed significant association with pulmonary function parameters, as carriers of the CC genotype have higher FVC, pre-FEV1, and post-PEFR, highlighting a better response to therapy. The previous studies declared only the relation between HSP70 levels and pulmonary function tests and found that elevated HSP70 levels in the sputum and plasma were negatively correlated with FEV1 and FEV1/FVC in asthma [8,52]. Additionally, circulating HSP70 was elevated and negatively correlated with FEV1, FEV1, and FEV1/FVC in COPD, a common chronic airway disease such as asthma [52,61]. HSP70 deficiency was also related to idiopathic pulmonary fibrosis, a common restrictive lung disease characterized by impaired pulmonary function, and it has been proposed as a potential biomarker for lung lifespan [62]. Altogether, HSP70 could be a promising biomarker candidate for chronic lung disease theranostics monitoring.

Although the current study was the first to address the relationship of *HSPA1B* rs2763979 with asthma, some limitations should be considered: First, the sample was relatively small-sized. Second, both patients and controls were enrolled in one hospital, so selection bias could not be avoided. Third, gene expression levels should have been determined, but insufficient collected sample volume interfered. Fourth, it is noteworthy to consider using genetic matching techniques to reduce selection bias rather than dependence on propensity matching analysis, as proposed by several scientists [63,64,65]. Therefore, it is recommended to replicate the work in multi-center, larger-scale studies in different ethnic groups. Moreover, including other SNPs related to *HSP70* by applying robust genetic matching with functional and mechanistic analyses will be helpful.

## 5. Conclusions

This study unequivocally reported that *HSPA1B* rs2763979 was associated with asthma prognosis regarding favorable pulmonary function parameters. This work may provide the impetus for future multi-centric and mechanistic research to support our findings and comprehend the underlying molecular pathways.

## Figures and Tables

**Table 1:** Baseline characteristics of the study groups.

Demographic Data		Unmatched Cohorts			Matched Cohorts		
		Controls	Asthma	*p*-Values	Controls	Asthma	*p*-Values
Total Number		218	90		71	71	
Mean age, years	Mean ± SD	9.2 ± 3.05	9.76 ± 2.9	0.17	9.76 ± 3.2	9.6 ± 3.03	0.81
Age categories, %	6–11	174 (79.8)	68 (75.6)	0.44	51 (71.8)	52 (73.2)	0.85
	12–18	44 (20.2)	22 (24.4)		20 (28.2)	19 (26.8)	
Sex	Female	102 (46.8)	45 (50)	0.62	32 (45.1)	33 (46.5)	0.86
	Male	116 (53.2)	45 (50)		39 (54.9)	38 (53.5)	
Residence	Urban	107 (49.1)	59 (65.6)	**0.009**	46 (64.8)	44 (62)	0.86
	Rural	111 (50.9)	31 (34.4)		25 (35.2)	27 (38)	
BMI percentile	<85th	136 (62.4)	45 (50)	0.09	31 (43.7)	36 (50.7)	0.33
	<95th	81 (37.2)	45 (50)		27 (38)	28 (39.4)	
	≥95th	1 (0.5)	0 (0)		13 (18.3)	7 (9.9)	
Pubertal status	Negative	136 (62.4)	45 (50)	**0.045**	39 (54.9)	37 (52.1)	0.86
	Positive	82 (37.6)	45 (50)		32 (45.1)	34 (47.9)	
Tanner stage	Stage 1	138 (63.3)	47 (52.2)	0.091	40 (56.3)	38 (53.5)	0.53
	Stage 2	23 (10.6)	19 (21.1)		7 (9.9)	14 (19.7)	
	Stage 3	32 (14.7)	10 (11.1)		10 (14.1)	8 (11.3)	
	Stage 4	15 (6.9)	9 (10)		10 (14.1)	7 (9.9)	
	Stage 5	10 (4.6)	5 (5.6)		4 (5.6)	4 (5.6)	
FH of asthma	Negative	202 (92.7)	65 (72.2)	**<0.001**	60 (84.5)	60 (84.5)	1.0
	Positive	16 (7.3)	25 (27.8)		11 (15.5)	11 (15.5)	

Values are presented as numbers (percentage) and mean ± standard deviation. BMI, body mass index; pubertal status, sexual maturation, and secondary sexual characteristics were assessed using the “sexual maturity rating stages” by Marshall and Tanner [20]; FH, family history. Chi-square and Student t-tests were used. Bold values are statistically significant at *p* < 0.05.

**Table 2:** Genotype and allele frequencies of *HSPA1B* rs2763979 variant.

Characteristic	Levels	Total	Controls	Patients	*p*-Values
Genotypes					
	T/T	113 (37)	92 (42)	21 (23)	**0.008**
	C/T	169 (55)	109 (50)	60 (67)	
	C/C	26 (8)	17 (8)	9 (10)	
Allele					
	T allele	395 (64)	293 (67)	102 (57)	**0.006**
	C allele	221 (36)	143 (33)	78 (43)	

Data values are shown as numbers (%). Chi-square (χ^2^) for trends was used. The allele frequency within each group was determined as the number of occurrences of an individual allele divided by the total number of alleles. Bold values indicate significance at *p* <0.05.

**Table 3:** Genetic association models for asthma risk.

Model	Genotype	Controls (N = 218)	Patients (N = 90)	OR (95%CI)	*p*-Values
Codominant	T/T	92 (42.2%)	21 (23.3%)	1	
	C/T	109 (50%)	60 (66.7%)	2.75 (1.46–5.18)	**0.003**
	C/C	17 (7.8%)	9 (10%)	3.35 (1.19–9.39)	**0.008**
Dominant	T/T	92 (42.2%)	21 (23.3%)	1	
	C/T–C/C	126 (57.8%)	69 (76.7%)	2.83 (1.52–5.25)	**<0.000**
Recessive	T/T–C/T	201 (92.2%)	81 (90%)	1	
	C/C	17 (7.8%)	9 (10%)	1.77 (0.70–4.48)	**0.240**
Overdominant	T/T–C/C	109 (50%)	30 (33.3%)	1	
	C/T	109 (50%)	60 (66.7%)	2.12 (1.20–3.74)	**0.008**
Log-additive	---	---	---	2.09 (1.32–3.30)	**0.001**

Data are presented as frequency (percentage). Logistic regression analysis was employed. OR (95% CI), odds ratio and confidence interval. Adjusted covariates were age, sex, residency, BMI, puberty, and tanner stage.

**Table 4:** Association of *HSPA1A* genotypes with demographic and clinicolaboratory data in patients with BA (N = 71 patients).

Characteristics		Total	C/C (N = 9)	C/T (N = 48)	T/T (N = 14)	*p*-Value
Demographics						
Age, years	Mean ± SD	10.6 ± 3.2	11 ± 3.6	10.6 ± 3.3	10.3 ± 3	0.72
	6–11 years	52 (73.2)	6 (66.7)	35 (72.9)	11 (78.6)	0.82
	12–18 years	19 (26.8)	3 (33.3)	13 (27.1)	3 (21.4)	
Sex	Female	33 (46.5)	3 (33.3)	24 (50)	6 (42.9)	0.63
	Male	38 (53.5)	6 (66.7)	24 (50)	8 (57.1)	
Residency	Rural	44 (62)	6 (66.7)	30 (62.5)	8 (57.1)	0.89
	Urban	27 (38)	3 (33.3)	18 (37.5)	6 (42.9)	
Family history	Negative	60 (84.5)	7 (77.8)	40 (83.3)	13 (92.9)	0.58
	Positive	11 (15.5)	2 (22.2)	8 (16.7)	1 (7.1)	
Body mass index, %	<85th percentile	36 (50.7)	2 (22.2)	27 (56.3)	7 (50)	0.20
	<95th percentile	28 (39.4)	5 (55.6)	16 (33.3)	7 (50)	
	≥95th percentile	7 (9.9)	2 (22.2)	5 (10.4)	0 (0)	
Pubertal status	Negative	37 (52.1)	4 (44.4)	27 (56.3)	6 (42.9)	0.60
	Positive	34 (47.9)	5 (55.6)	21 (43.8)	8 (57.1)	
Clinical presentation						
Age at onset, years	Mean ± SD	3.5 ± 2	5.7 ± 2.1	3.4 ± 2	3.1 ± 1.6	0.89
	<3 years	37 (52.1)	4 (44.4)	25 (52.1)	8 (57.1)	0.84
	>3 years	34 (47.9)	5 (55.6)	23 (47.9)	6 (42.9)	
Duration, years	Mean ± SD	7.1 ± 2.9	5.3 ± 2.1	7.2 ± 2.9	7.2 ± 3.1	0.76
Asthma phenotype	Atopic asthma	55 (77.5)	5 (55.6)	39 (81.3)	11 (78.6)	0.11
	Non-atopic	6 (8.5)	1 (11.1)	3 (6.3)	2 (14.3)	
	Exercise-induced	9 (12.7)	3 (33.3)	6 (12.5)	0 (0)	
	Aspirin-sensitive	1 (1.4)	0 (0)	0 (0)	1 (7.1)	
Symptoms	Cough	69 (97.2)	9 (100)	47 (97.9)	13 (92.9)	0.52
	Dyspnea	40 (56.3)	5 (55.6)	27 (56.3)	8 (57.1)	1.00
	Sputum	40 (56.3)	3 (33.3)	29 (60.4)	8 (57.1)	0.32
	Tightness	46 (64.8)	5 (55.6)	30 (62.5)	11 (78.6)	0.45
	Wheezes	59 (83.1)	5 (55.6)	42 (87.5)	12 (85.7)	0.06
Triggering factors	Allergen sensitization	44 (62)	4 (44.4)	31 (64.6)	9 (64.3)	0.51
	Animal	17 (23.9)	1 (11.1)	13 (27.1)	3 (21.4)	0.57
	Food allergy	23 (32.4)	0 (0)	20 (41.7)	3 (21.4)	**0.031**
	Dust	20 (28.2)	1 (11.1)	15 (31.3)	4 (28.6)	0.47
	Pollen	16 (22.5)	3 (33.3)	9 (18.8)	4 (28.6)	0.53
	Exercise	47 (66.2)	5 (55.6)	33 (68.8)	9 (64.3)	0.73
	Cold air	36 (50.7)	4 (44.4)	25 (52.1)	7 (50)	0.91
	Aspirin	15 (21.1)	0 (0)	11 (22.9)	4 (28.6)	0.23
	Conjunctivitis	23 (32.4)	4 (44.4)	16 (33.3)	3 (21.4)	0.50
	Sinus–ear infection	31 (43.7)	5 (55.6)	22 (45.8)	4 (28.6)	0.39
	Perfume	27 (38)	3 (33.3)	20 (41.7)	4 (28.6)	0.64
Risk factors	RTI	44 (62)	6 (66.7)	28 (58.3)	10 (71.4)	0.64
	Seasonal	4 (5.6)	1 (11.1)	3 (6.3)	0 (0)	1.00
	Emotion stress	23 (32.4)	2 (22.2)	17 (35.4)	4 (28.6)	0.70
	Smoking	46 (64.8)	6 (66.7)	33 (68.8)	7 (50)	0.43
	Rhinitis	26 (36.6)	3 (33.3)	19 (39.6)	4 (28.6)	0.74
	Hives	22 (31)	4 (44.4)	17 (35.4)	1 (7.1)	0.09
	Eczema	19 (26.8)	4 (44.4)	13 (27.1)	2 (14.3)	0.28
	Anaphylaxis	15 (21.1)	3 (33.3)	11 (22.9)	1 (7.1)	0.28
Comorbidities	Negative	36 (50.7)	3 (33.3)	24 (50)	9 (64.3)	0.35
	Positive	35 (49.3)	6 (66.7)	24 (50)	5 (35.7)	
Disease severity						
Daytime symptoms (>2 weeks)	Negative	37 (52.1)	5 (55.6)	26 (54.2)	6 (42.9)	0.74
	Positive	34 (47.9)	4 (44.4)	22 (45.8)	8 (57.1)	
Night awakening	Negative	63 (88.7)	9 (100)	43 (89.6)	11 (78.6)	0.27
	Positive	8 (11.3)	0 (0)	5 (10.4)	3 (21.4)	
Activity limitations	Negative	52 (73.2)	7 (77.8)	35 (72.9)	10 (71.4)	0.94
	Positive	19 (26.8)	2 (22.2)	13 (27.1)	4 (28.6)	
Asthma severity	Mild	33 (46.5)	5 (55.6)	19 (39.6)	9 (64.3)	0.24
	Moderate	28 (39.4)	3 (33.3)	23 (47.9)	2 (14.3)	
	Severe	10 (14.1)	1 (11.1)	6 (12.5)	3 (21.4)	
Airway hyper-responsiveness	Normal	32 (45.1)	6 (66.7)	23 (47.9)	3 (21.4)	0.38
	Borderline	22 (31)	1 (11.1)	15 (31.3)	6 (42.9)	
	Mild/moderate	16 (22.5)	2 (22.2)	9 (18.8)	5 (35.7)	
	Severe	1 (1.4)	0 (0)	1 (2.1)	0 (0)	
Laboratory data						
High IgE level	Positive	26 (36.6)	2 (22.2)	17 (35.4)	7 (50)	0.38
Eosinophilia	Positive	9 (12.7)	2 (22.2)	5 (10.4)	2 (14.3)	0.71
Total IgE (IU/mL)	Median (IQR)	80 (24–126)	75 (40–162.5)	80 (25–126)	100 (20–123)	0.94
Eosinophil Count (×10^6^/L)	Median (IQR)	125 (32–245)	32 (22–506)	145 (50–245)	120 (30–235)	0.90

Values are shown as number (%), mean ± standard deviation (SD), or median (interquartile range). Two-sided Chi-square, one-way ANOVA, and Kruskal–Wallis tests were used. Bold values indicate significance at *p* < 0.05.

**Table 5:** Association of *HSPA1A* genotypes with demographic and clinicolaboratory data in patients with BA (N = 71 patients).

Characteristics		Total	C/C (N = 9)	C/T (N = 48)	T/T (N = 14)	*p*-Value
Management						
Reliever use	Negative	47 (66.2)	7 (77.8)	29 (60.4)	11 (78.6)	0.33
(>2 weeks)	Positive	24 (33.8)	2 (22.2)	19 (39.6)	3 (21.4)	
Asthma Control	Well controlled	26 (38.2)	5 (55.6)	18 (40)	3 (21.4)	0.40
	Partly controlled	34 (50)	3 (33.3)	21 (46.7)	10 (71.4)	
	Uncontrolled	8 (11.8)	1 (11.1)	6 (13.3)	1 (7.1)	
Therapy Level	Step 1	18 (25.4)	4 (44.4)	10 (20.8)	4 (28.6)	0.60
	Step 2	15 (21.1)	1 (11.1)	9 (18.8)	5 (35.7)	
	Step 3	11 (15.5)	1 (11.1)	9 (18.8)	1 (7.1)	
	Step 4	22 (31)	2 (22.2)	16 (33.3)	4 (28.6)	
	Step 5	5 (7)	1 (11.1)	4 (8.3)	0 (0)	
Pulmonary function test						
FVC (% predicted)	Mean ± SD	77.8 ± 6.9	86.3 ± 7.4	77.7 ± 6.1	75.7 ± 7.2	**0.021**
Pre-FEV_1_ (% predicted)	Mean ± SD	55.7 ± 7.9	60.7 ± 12.9	54.9 ± 7.6	56.1 ± 7.5	**0.021**
Post-FEV_1_ (% predicted)	Mean ± SD	76.9 ± 7.8	81 ± 13	76.1 ± 7.6	77.5 ± 7.2	0.052
Post-PEFR (% predicted)	Mean ± SD	75.3 ± 12.6	77.7 ± 27.4	74.3 ± 11.7	77.1 ± 10.9	**0.044**
BDRBASE (% predicted)	Mean ± SD	39 ± 6.8	34.7 ± 8.2	39.4 ± 6.4	39.1 ± 7.5	0.17

Values are shown as numbers (%) or mean ± standard deviation (SD). Two-sided Chi-square and one-way ANOVA tests were used. FVC: forced vital capacity (pre-test value); pre/post-FEV1: forced expiratory volume in 1 s before/after bronchodilation; post-PEFR: peak expiratory flow rate after bronchodilation; BDRBASE, change in FEV1 as a percent of baseline FEV1 [((post-BDFEV1 − pre-BDFEV1)/pre-BDFEV1) × 100]. Bold values indicate significance at *p* < 0.05.

## Data Availability

All generated data in this study are included in the article.

## Supplementary Materials

The following supporting information can be downloaded at: https://www.mdpi.com/article/10.3390/genes13122391/s1, Table S1: Characteristics of the included studies in the meta-analysis.
