# Supplementary material for: Diagnostic and Prognostic Risk Assessment of Heat Shock Protein HSPA1B rs2763979 Gene Variant in Asthma
Source: Genes (Basel). 2022 Dec 16;13(12):2391. doi: 10.3390/genes13122391 (PMC9778050; doi:10.3390/genes13122391)
Supplement: Supplementary file 1 [file genes-13-02391-s001.zip › genes-1983935-supplementary.pdf]

Table S1. Characteristics of the included studies in the meta-analysis

First author [Ref.]	Year	Country	Ethnicity	Disorder	Genotyping Method	Patients N				Controls* N			
						N	T/T	T/C	C/C	N	T/T	T/C	C/C
Change et al. [44]	2021	Taiwan	Asian	Age related hearing impairment	TaqMan genotyping assay	146	11	50	82	146	20	72	50
Wang et al. [14]	2021	Chinese	Asian	Coronary artery disease	PCR–ligase detection reaction	271	45	122	104	113	16	60	37
Kowalczyk et al. [45]	2020	Poland	Caucasian	Paranoid schizophrenia	PCR-RFLP	377	44	162	171	524	63	224	237
Ambrocio-Ortiz et al [16]	2020	Mexico	Caucasian	Chronic obstructive lung disease	TaqMan genotyping assay	1103	321	624	158	424	89	244	91
Dhamodharan [17]	2017	South India	Asian	Diabetic nephropathy	PCR-RFLP	460	186	397	107	256	37	107	49
Li et al. [41]	2017	China	Asian	Noise induced hearing loss	SNPscan multiplex SNP genotyping	286	49	133	104	286	31	139	116
Chang et al. [46]	2011	Taiwan	Asian	Noise induced hearing loss	TaqMan genotyping assay	27	0	9	18	322	19	124	179
Guo et al. [43	2011	China	Asian	Lung cancer	TaqMan genotyping assay	1152	94	457	601	1152	84	457	611

N: Number, PCR-RFLP: polymerase chain reaction- Restriction fragment length polymorphism. *All enrolled controls in the included studies were population-based controls.
